# Health care provider time in public primary care facilities in Lima, Peru: a cross-sectional time motion study

**DOI:** 10.1186/s12913-021-06117-9

**Published:** 2021-02-06

**Authors:** Hannah H. Leslie, Denisse Laos, Cesar Cárcamo, Ricardo Pérez-Cuevas, Patricia J. García

**Affiliations:** 1 Department of Global Health and Population, Harvard TH Chan School of Public Health, 677 Huntington Ave, Boston, MA USA; 2 School of Public Health, Universidad Peruana Cayetano Heredia, Lima, Peru; 3 Inter-American Development Bank, Lima, Peru; 4 Division of Social Protection and Health, Jamaica Country Office, Inter-American Development Bank, 6 Montrose Road, Kingston, Jamaica

**Keywords:** Peru, Primary health care, Absenteeism, Time motion, Health information systems

## Abstract

**Background:**

In Peru, a majority of individuals bypass primary care facilities even for routine services. Efforts to strengthen primary care must be informed by understanding of current practice. We conducted a time motion assessment in primary care facilities in Lima with the goals of assessing the feasibility of this method in an urban health care setting in Latin America and of providing policy makers with empirical evidence on the use of health care provider time in primary care.

**Methods:**

This cross-sectional continuous observation time motion study took place from July – September 2019. We used two-stage sampling to draw a sample of shifts for doctors, nurses, and midwives in primary health facilities and applied the Work Observation Method by Activity Timing tool to capture type and duration of provider activities over a 6-h shift. We summarized time spent on patient care, paper and electronic record-keeping, and non-work (personal and inactive) activities across provider cadres. Observations are weighted by inverse probability of selection.

**Results:**

Two hundred seventy-five providers were sampled from 60 facilities; 20% could not be observed due to provider absence (2% schedule error, 8% schedule change, 10% failure to appear). One hundred seventy-four of the 220 identified providers consented (79.1%) and were observed for a total of 898 h of provider time comprising 30,312 unique tasks. Outpatient shifts included substantial time on patient interaction (110, 82, and 130 min for doctors, nurses, and midwives respectively) and on paper records (132, 97, and 141 min) on average. Across all shifts, 1 in 6 h was spent inactive or on personal activities. Two thirds of midwives used computers compared to half of nurses and one third of doctors.

**Conclusions:**

The time motion study is a feasible method to capture primary care operations in Latin American countries and inform health system strengthening. In the case of Lima, absenteeism undermines health worker availability in primary care facilities, and inactive time further erodes health workforce availability. Productive time is divided between patient-facing activities and a substantial burden of paper-based record keeping for clinical and administrative purposes. Electronic health records remain incompletely integrated within routine care, particularly beyond midwifery.

**Supplementary Information:**

The online version contains supplementary material available at 10.1186/s12913-021-06117-9.

## Background

Efficiency – the provision of health services to maximize existing resources and minimize waste – is a key attribute of a high-quality health system [1–3]. From a system perspective, achieving efficiency requires delivery of the necessary services at the level most appropriate to provide quality care; within service delivery, efficiency depends upon the effective translation of available resources into value for patients [3]. Primary care is particularly critical to health system efficiency: primary care services that meet population needs and provide effective primary and secondary prevention result in better and more equitable population health benefits than higher cost specialty care [4, 5]. Especially in resource-constrained settings, the absence of trusted and high-quality primary care has led to avertable morbidity and mortality and wasteful or even harmful overuse of specialist care [2, 6, 7]. Evidence on the delivery of primary care services to meet population health needs in the face of constrained resources is needed given the ongoing effects of the Covid-19 pandemic on both health services and health financing.

As in many Latin American countries, the health system in Peru faces a growing burden of noncommunicable diseases such as cardiovascular disease and diabetes, particularly in Lima, home to nearly 11 million people, even as total life expectancy continues to increase [8, 9]. Primary care services are struggling to provide the continuity of care required to prevent and manage illness over time and are perceived to lack the resources and support required for high quality care [10]. Deficits and delays in primary care services result in the majority of the population avoiding routine preventive medical care [10], in frequent bypassing of primary care for pharmacies [11], and, for users of primary care in Lima, in an difficulty obtaining medications prescribed at the visit and dissatisfaction with long wait times [12, 13].

The health workforce is a key constraint in Peru, where there were 13 doctors per 10,000 individuals in 2016, one of the lowest levels in Latin America, and 24 nurse-midwives per 10,000 [14]. The public primary care system in particular has seen challenges in recruiting and retaining medical and nursing trainees [15]; the average health care provider in the public sector in Lima is 45 years old, 5 years older than the average provider in the private sector [16]. Since by law health care provider shifts are limited to 6 h and a maximum of 36 h per week, dual practice is common, with three in four private physicians near Lima working at least one other position, most commonly as a public provider [17]. Since 2013, the Ministry of Health (MOH) – which provides health services to over two thirds of the population [18] - has engaged in health system reform aiming to expand access to services and strengthen the inputs to health services as a pathway to universal health coverage [19, 20]. Increased financing and greater human resources for primary care were a key pillar of this effort [19]. The MOH has also looked to information technology to improve service delivery, although to date health information systems have been deployed in a largely fragmented manner: in 2017, the MOH had over 300 separate non-interoperable systems. Efforts to develop an electronic medical record (EMR) have been underway since 2010, starting with antenatal care [21, 22] and expanding in 2016 to include additional modules for primary care services. Limited research to date has addressed the delivery of primary care services in light of these reforms.

Informing and evaluating health system performance requires measures that capture the productivity of primary care services, including provider use of time, effectiveness, and value to patients. While there are cross-national comparisons of patient experience in Latin America [23] and some evidence specific to Peru on patient satisfaction with care in public hospitals [24], little information is currently available on the productivity of the health workforce in primary care delivery, particularly in the context of expanding health technology systems. The dominant method for studying health care providers’ use of time in health services is the time motion study, a method of direct observation [25]. Most such studies to date have been conducted in high-income countries [26]; there is a dearth of information available to assess and compare health care worker productivity and inform health system reforms in middle-income countries. The aims of the present study are to demonstrate the feasibility of a time motion study in urban primary care facilities and to describe the use of time by health care providers per shift in Lima, including the frequency of health information technology use within the daily practice of primary care.

## Methods

The study protocol with full details is provided in the Supplemental Materials, as is the STROBE checklist for cross-sectional studies.

### Setting

We conducted a continuous observation time motion assessment as a cross-sectional study at two tiers of primary care facilities in Lima, Peru. The MOH manages most of the public primary care facilities in the country; these facilities are divided into tiers from I-1 through I-4, where I-4 is the highest capacity. In Supplemental material, Table S1 provides details on the tiers of primary care service delivery. We studied the more commonly used I-3 and I-4 facilities, which are staffed by a minimum of a doctor, nurse midwife, nurse technician, and laboratory technician. These facilities provide outpatient and preventive health services; they are required to have laboratory services. I-4 facilities may additionally include maternity wards and employ pediatricians and gynecologists. The 209 I-3 and I-4 facilities in Lima employed 3321 doctors, nurses, and midwifes at the time of the study.

### Sampling

We conducted two-stage sampling, first at the facility level and then at the provider level. Facilities were randomly sampled from the database provided by the MOH within strata of facility tier (I-3, I-4). Facility directors at sampled facilities were approached for consent. Those consenting were asked to provide updated shift schedules for the subsequent week(s). One provider shift was selected at random for each type of provider (doctors, nurses and midwives) from these schedules. In cases where providers could not be found or declined to participate, a new shift was sampled at random for the following 7 days of enumerator availability. Neither providers nor facility directors were offered incentives to participate.

We calculated a sample size large enough to provide ±3% precision for the primary outcome of time spent on direct patient care. Drawing from existing literature on time-motion observations [25, 27–29], we estimated standard deviation in this outcome up to 10%, which resulted in a target sample size of 54 providers per type. To account for potential non-response, particularly at small facilities with one or two providers per type, we sampled 60 facilities. We calculated sampling probability for each observation based on the number of providers of each type employed at the facility during the month the observation took place. Consideration of clustering by facility was not necessary as a single provider of each type was sampled per facility; analyses were conducted within provider types. We used administrative data available on all health care workers at MOH facilities in Lima to compare the sample achieved against the target population for potential selection bias [16]. We assessed overall feasibility of the time-motion study based on capacity to capture complete and usable data for all provider types from a representative sample of primary care facilities.

### Instrument

We used the Work Observation Method by Activity Timing (WOMBAT, Australian Institute for Health Innovation, Macquarie University, North Ryde, NSW Australia) software program to assess provider time use [30]. This tool has been used in multiple countries and health system settings [31, 32], but not to our knowledge in Latin America. Using a tablet device, observers start a record for each task observed and classify it across three dimensions: activity, communication, and location. Tasks are ended once the task is complete, with time recorded exactly at start and end. For each dimension we defined major categories and subcategories based on existing literature on types of activities health professionals perform, adapted for the study context. Sub-categories were mutually exclusive; however, observers were able to designate interruptions and multitasking as they occurred.

### Data collection

The team of 3 supervisors and 10 field workers was selected in June 2019; all team members had experience with primary health services as providers or trainees. Team members were trained for 5 days on research ethics, study procedures, and the use of the WOMBAT program and recognition of activity types using publicly available standardized patient cases. An additional facility was selected in the original sample to provide a pilot site; following consent from the facility director, 8 individual health care providers were selected, with each assigned 2 field workers for piloting. Based on the pilot, activity categories and sub-categories were refined to better reflect the provider tasks, including creating a separate template for doctors with a small number of clinical activities that nurses and midwives did not perform. The final tools are shown (in Spanish) in the Supplement, Figure S1 and S2.

The full cross-sectional study was conducted from July to September of 2019, with brief interruptions due to changes in health worker schedules for national holidays and a national health worker strike. During the week each facility was scheduled for assessment, study field workers visited the facilities unannounced 30 min prior to the scheduled start of the shifts and attempted to locate the selected provider for up to 2 h following the start of shift. If providers were located and consented to participate, the field worker was instructed to apply the observation protocol using WOMBAT until the scheduled end of the shift or the time the provider left the facility for non-work with no intention of returning. In the event the provider left the facility for work purposes (e.g. transfer patients in ambulance, home visits, etc.), observers requested permission to follow and if denied, waited for the provider to return until the end of the scheduled shift. If a provider or patient preferred for the observer not to be present during any clinical task, the observer waited nearby and recorded the activity as unobserved. Observers recorded the type of shift from the schedule. We grouped shifts into broad categories of outpatient (outpatient and triage), inpatient/urgent care (emergency, obstetric center, and inpatient urgent care) and other (administrative, community outreach, a mix of multiple responsibilities).

### Measures

We grouped activities from the time motion observation into work types based on previous use of the WOMBAT tool for time-motion observations [30]. Classifications (Table 1) were made by consensus among three authors and reviewed for consistency by the senior author PJG.

**Table 1:** Classifications of Activity Types from Time Motion Observation

Activity type	Definition	Example codes
Direct patient care	Procedures, communicating with a patient or family member	Procedures: Physical exam / signs Procedures: Clinical history
Indirect patient care	Reading patient history, ordering tests, washing hands, cleaning up after procedure, retrieving information	Paper-read: Clinical history Computer-read: Clinical history Search: Preparation of materials and supplies Paper-write: Order tests
Medication	Finding orders; prescribing, preparing or administering medication	Search: Order Phone: Prescription Computer-read: Prescriptions Paper-write: Prescription
Documentation	Recording patient information on paper or computer	Paper-write: Clinical history Computer-write: Patient registries
Professional communication	Work-related discussions, including requesting consults, presenting or handing over patients	Computer-write: Referral / consult Communication: Health workers, about work
Administrative	Staff meetings, administrative paperwork, coordination of staff schedules or activities	Paper-write: FUA Paper-read: FUA Search: Registry
Transit	Movement between patients and between tasks	Movement: Inside health facility Movement: Outside health facility – home visit
Education	Attending education sessions as teacher or student	Paper-read: References (books or others) Communication only: Listening to instruction
Personal / social	Any social or personal activity or discussion	Personal: Bathroom Phone: Applications – personal Personal: Leisure time
Inactive	No activity, including time from departure to end of shift for providers leaving early	Other: Inactivity

*FUA* Formato Unico de Atención (Single Care Form)

Periods without observation or with insufficient detail available (such as “Computer - read: Other) were classified as Other. Minutes from departure to the scheduled end of 6-h daytime shifts counted towards inactive time if providers departed for non-work activities before the shift concluded. Time from the scheduled start of shift to the beginning of observations was not eligible for analysis since providers had to provide consent prior to the observation starting.

We additionally defined 3 overarching activity classifications: any patient interaction, any documentation with paper, and any documentation with computers. These categories are not mutually exclusive as the activities above are. They are defined as:

- Patient care and communication: direct patient care and all time communicating with patient and family, even if other activities such as procedures or documentation were happening at the same time.
- Paper records: all activities conducted with paper records, including reading, and writing patient information, except for printing.
- Computerized records: all activities conducted with electronic resources, including reading, or writing electronic records, and printing.

We calculated proportion of time per activity out of actual minutes from the start of observation to end of observation or end of shift, whichever was later. We also defined a binary indicator of early departure as observations ending more than 20 min before scheduled end of daytime shifts.

We assessed the time motion data for data quality. First, we identified all individual tasks with duration over an hour as potentially erroneous (47 tasks within 40 providers). We reviewed the start and end time of these tasks, their timing within the observation, and the content and location of tasks within the pattern of provider activity for evidence that a task had been started or continued in error, such as overlapping tasks in different locations or with conflicting activities (e.g., no communication and communication with patient). We replaced as missing those tasks that met three criteria: they were the first or second task the observer recorded after beginning the observation, they lasted over 100 min, and they overlapped with at least one task that conflicted in content or location.

### Analysis

We report descriptive statistics of the health facilities and individual providers selected for participation and those ultimately enrolled in the study. Differences between participating and non-participating providers were assessed using the Chi Square test for categorical data and the Kruskal-Wallis test for differences in distribution for continuous variables.

We calculated sampling weights as the inverse probability of selection, scaled to sum to the observed number of providers per cadre. Analysis accounts for stratified sampling and probability weights. We present descriptive statistics of the total observations, including duration, raw number of tasks observed, and proportion of providers departing early.

We summarized the number of providers performing each category of activity and the mean minutes spent per activity by provider cadre and shift type (outpatient, inpatient/urgent, other). We tested the significance of differences in activities by shift type using design-based F tests for proportion of providers performing the activity and linear regression for time per activity in order to incorporate sample strata and weights. Although we did not capture time spent by unique patient in the observations, we calculated and plotted time spent consecutively on patient-facing activities as an approximation of time per patient. We calculated the proportion of time spent on individual activities and summarized these graphically by provider cadre. We quantified time spent transcribing records as an indication of potential inefficiencies in health facility operation. Finally, we compared the time spent on direct patient care and the time spent on documentation for providers observed to use computers at all and those not observed using computers using linear regression. Raw data were exported to comma delimited files; analysis was performed using Stata 16.0 (Stata Corp, College Station, Texas).

## Results

### Study sample

Of the initial sample of 60 health facilities (40 I-3, 20 I-4), one was excluded due to provision of mental health services only and one declined to participate. Two replacement facilities were sampled. A total of 275 providers were sampled for the study. Comparison to administrative data on all providers in the population (Supplement, Table S2) suggests composition of the sample was generally similar to the underlying population on characteristics such as age (sample average 46 years, population 49), contract type (sample 71% permanent, population 83%), and facility tier (sample 36% from I-4 facilities, population 40% I-4 facilities). As in the population data, nearly all nurses (93% in sample, 96% in population) and midwives (100% in sample, 96% in population) are female, while the split for doctors is closer to even (53% female in sample, 42% in population).

Provider characteristics by participation status are shown in Table 2. Of 275 sampled shifts in primary care facilities, 55 (20%) could not be observed: in 6 cases, the named provider did not work at that health facility, in 21 the provider had switched shifts since the weekly schedule was released, and in 28 cases the provider did not show up for a scheduled shift (unplanned absenteeism [33]). There were no statistically significant differences between these 55 cases and the 220 providers identified based on the demographics and contract information provided by the MOH.

**Table 2:** Characteristics of providers sampled for observation (unweighted *N* = 275)

	Not found	Refused/with-drew consent	Consented and observed	Total
	(n_1_ = 55)	n_2_ = 46)	(n_3_ = 174)	(n = 275)
	**Mean (SD)**	**Mean (SD)**	**Mean (SD)**	**Mean (SD)**
**Age (years)**	45.7 (9.4)	51.0 (9.7)	44.9 (10.5)	46.1 (10.4)
**Years worked in health center**	9.7 (8.7)	14.3 (10.0)	12.2 (10.3)	12.1 (10.0)
	**n (col. %)**	**n (col. %)**	**n (col. %)**	**n (col. %)**
**Facility tier**				
I-3	34 (62%)	25 (54%)	116 (67%)	175 (64%)
I-4	21 (38%)	21 (46%)	58 (33%)	100 (36%)
**Provider type**				
Doctor	19 (35%)	17 (37%)	59 (34%)	95 (35%)
Nurse	20 (36%)	14 (30%)	58 (33%)	92 (33%)
Midwife	16 (29%)	15 (33%)	57 (33%)	88 (32%)
**Gender**				
Male	10 (19%)	10 (22%)	29 (17%)	49 (18%)
Female	44 (81%)	36 (78%)	139 (83%)	219 (82%)
**Type of contract**				
Fixed term	8 (15%)	5 (11%)	30 (18%)	43 (16%)
Permanent	41 (76%)	40 (87%)	111 (65%)	192 (71%)
Short term	5 (9%)	1 (2%)	29 (17%)	35 (13%)

Of the 220 providers approached for participation, 174 (79.1%) consented and were observed for the duration of the shift: 59 doctors, 58 nurses, and 57 midwives. Among those found, those with permanent positions were more likely to decline consent (40 of 151, 26.5%) than fixed term employees (5 of 35, 14.3%) and employees on short-term flexible contracts (1 of 30 3.3%), *p* < 0.05. Those consenting were younger than those declining to participate (mean 44.9 years for consented individuals vs. 51.0 years for those declining to participate, p < 0.05).

### Time motion observations

A total of 898 h of provider time were observed, comprising 30,312 unique tasks; 22 tasks for 20 unique providers were replaced as missing following data quality assessment. Observations lasted a median of 5.3 h (interquartile range [IQR] 4.7–5.7 h). The median number of tasks observed per shift was 148 for nurses, 163 for midwives, and 187 for doctors. Figure S3 in the Supplement depicts the observations from starting minute to ending minute, with I-3 health facilities observations (*N* = 116) in Figure S3A and I4 (*N* = 58) in Figure S3B. Scheduled shift start times are shown as red lines. Eighteen percent of daytime shifts ended more than 20 min before the scheduled end. Early departures were more common in I-3 facilities than I-4 facilities, particularly for morning shifts (50% of I-3 shifts vs. 1.5% of I-4 shifts, design-based F test *p* < 0.05, compared to 27% of I-3 shifts and 13% of I-4 shifts with early departures in afternoons, *p* = 0.34). Early departures did not differ significantly by provider type or form of contract.

### Provider activities

Table 3 presents the frequency and duration of overall activities by type of shift and provider cadre. For all cadres, the majority of shifts observed were of individuals providing outpatient care, as expected at these primary care facilities. Patient interaction and use of paper records were nearly universal in this sample. Looking at patient-facing tasks as consecutive blocks, doctors on outpatient shifts spent an average of 3.3 min per patient interaction with 35 interactions per shift; inpatient doctors spent similar time but had only 15 interactions per shift. Nurses and midwives averaged 4 to 5 min per patient interaction and had 25 to 30 such interactions per shift (Supplement, Table S3). Overall, doctors performing outpatient shifts spent 110 min on average on patient-facing care and communication (30.6% of a full shift) and 132 min on paper records (37%), significantly more in both cases than did doctors on inpatient/urgent shifts (45 min of patient interaction [12.5% of a shift] and 64 min [17.8% of a shift] on paper records, respectively). Other doctor shifts involved very little patient contact and an average of 46 min on patient records [12.8%].

**Table 3:** Proportion of providers conducting activities and time spent on activities when observed

	Outpatient		Inpatient/urgent		Other		Total	
	Frequency	Duration (minutes) if done	Frequency	Duration (minutes) if done	Frequency	Duration (minutes) if done	Frequency	Duration (minutes) if done
	%	Mean (SD)	%	Mean (SD)	%	Mean (SD)	%	Mean (SD)
**A: Doctors**		(*N* = 49)		(*N* = 7)		(*N* = 3)		(*N* = 59)
Patient-facing care and communication	100%	110 (39)	100%	**45 (39)**	9%	**3**	95%	97 (47)
Use of paper records	98%	132 (51)	100%	**64 (48)**	100%	**46 (34)**	99%	115 (58)
Use of computerized records	34%	26 (22)	20%	**89**	45%	8 (14)	33%	31 (29)
**B: Nurses**		(*N* = 46)		(N = 5)		(N = 7)		(*N* = 57)
Patient-facing care and communication	99%	82 (58)	100%	110 (52)	100%	**45 (19)**	99%	86 (56)
Use of paper records	100%	97 (56)	100%	**48 (42)**	100%	**35 (38)**	100%	83 (57)
Use of computerized records	49%	35 (42)	62%	18 (23)	52%	44 (41)	52%	32 (38)
**C: Midwives**		(N = 46)		(*N* = 8)		(N = 3)		(N = 57)
Patient-facing care and communication	97%	130 (79)	100%	93 (74)	100%	81 (38)	98%	115 (77)
Use of paper records	100%	141 (62)	100%	**55 (22)**	100%	114 (35)	100%	110 (65)
Use of computerized records	65%	35 (28)	66%	61 (36)	13%	**7**	63%	44 (32)

Frequency is the proportion of providers observed to do each activity at least once; duration is mean minutes per activity among providers observed in that activity. Standard deviations not calculated for cells with a single observation

Outpatient includes consultations and triage. Hospitalization includes emergency, hospitalization, and obstetric center shifts. Other includes administrative work, home visits, and shifts with multiple classifications

Bold highlight indicates significant difference (p < 0.05) from outpatient shifts for that cadre

Nurses spent an average of 82 min with patients per outpatient shift (22.8%) and 97 min on paper records (26.9%); inpatient shifts included 110 min (30.6%) with patients and 48 min on paper records (13.3%), significantly less than outpatient shifts. Other shifts involved shorter durations of patient time and paper records.

Midwives on outpatient shifts spent the most time of any providers on patient-facing care (130 min, 36.1%) and paper records (141 min, 39.2%); inpatient shifts included 93 min of patient care (one-fourth of a shift) and 55 min with paper records (15.2%), significantly less than outpatient. Unlike the other cadres, other shifts for midwives did not differ in terms of patient time or record time.

One third of doctors, half of nurses, and two thirds of midwives were observed to use computers. Among those using computers, midwives on inpatient shifts were observed to do so for more than an hour (61 min) per shift, as were the few doctors on inpatient shifts who used computers at all (89 min).

**Fig. 1:**
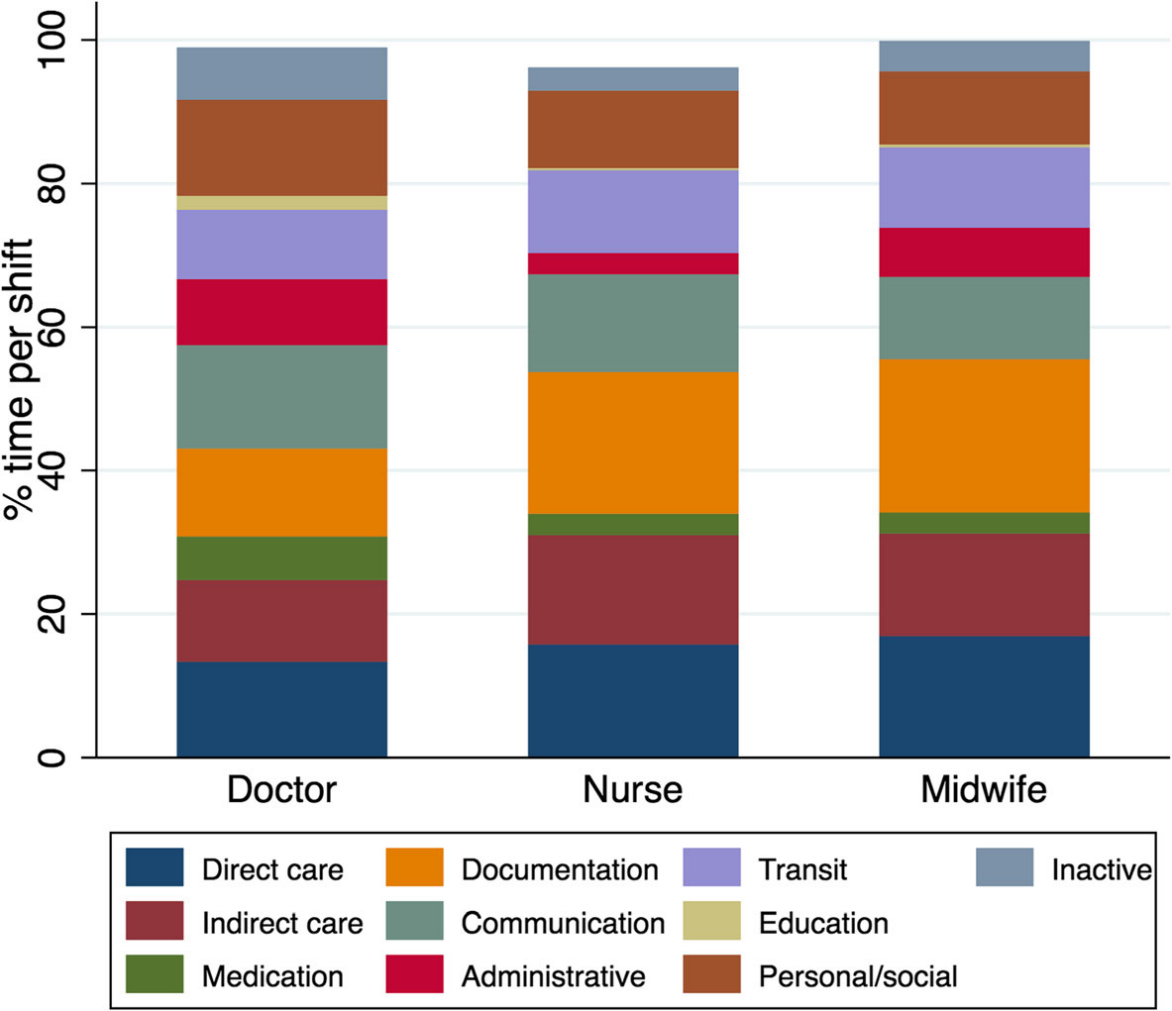
Proportion of provider time spent by activity category

We included 10 mutually exclusive categories for this analysis of the proportion of shift spent in each category (Fig. 1; unobserved time is left blank). Approximately 15% of all provider time was spent on direct care (SD 7.8% for doctors to 11.5% for nurses), with 11% on indirect care for doctors and 14–15% for midwives and nurses. Doctors spent almost as much time on documentation as they did on direct care, while nurses and midwives spent 20% of their shifts on documentation, more than they did on direct care. Administrative tasks took up the most time for doctors of the three provider types, a mean of 9% compared to 3% for nurses and 7% for midwives. Doctors spent over 20% of their time on either personal activities or inactive compared to 14% for nurses and midwives.

Detail on the specific tasks that providers conducted are shown in Table S4 in the Supplement. Time spent documenting patient care on patient records was the single most common activity on outpatient shifts, consuming between 11 and 19% of provider time. This time is exclusive of administrative paperwork such as the FUA, which was a substantial use of time among doctors and nurses completing outpatient shifts as well. Shifts focused on inpatient care included more substantial personal time, between 16 and 40% of total time. While the median provider spent only 5–8% of each shift on any personal time, individual providers devoted half to two thirds of daytime shift hours to personal time, resulting in an average of 10–13% of each shift spent on personal time (Figure S4, Supplement). Taking into account personal time as well as inactive time, the average provider would lose nearly an entire shift (6 h) per 36-h week of work to time spent not working.

### Time spent on documentation and health information

Time documenting patient information on computers included transcription of previously written hard copies: of a total of 32.9 h of computerized documentation observed, 6.4 h were spent on transcription. Most of this effort was among midwives (4 h).

In the Supplement, Table S5 presents results on how providers who did and did not use computers spent time on shifts, excluding administrative shifts (*N* = 4). There were few significant differences in time spent on direct patient care or documentation across providers: doctors observed using computers spent 4% less time on direct care and 3% more time on documentation, although these differences are not significant. Nurses’ time in direct care or documentation did not differ by computer use. Midwives using computers spent 5% more time on direct care (*p* < 0.10) and 12% more time on documentation (*p* < 0.05) than those not using computers.

## Discussion

### Summary of findings

This study establishes the feasibility of performing a study of time use by primary care providers in Lima and can provide a template for similar studies in urban areas in Latin America to generate comparative data. Despite challenges such as a health provider strike during the study and frequent changes to scheduled shifts, nearly all facilities and most identified providers consented to participate. The field team was able to observe consenting providers for the duration of their shift and to obtain data with few evident errors. The findings demonstrate that at the time of this assessment in mid-2019, 10% of scheduled shifts were unplanned absences; those present at the facilities spent on average 5 h of their 6-h shifts working, with remaining time inactive or on personal activities. Providers of outpatient care spent most of their working time interacting with patients and on paper records, spending 1.5–2 h on each activity on average across cadres. Computer use was less frequent than paper record-keeping and was less common for doctors and nurses than for midwives, indicating that electronic medical records remained incompletely integrated into primary care practice in Lima, particularly outside of maternal health care services.

### Practice and policy implications

Research in the early 2000s found that primary care providers were absent from clinics in Peru up to 25% of the time, with greater absenteeism among doctors and permanent (non-contract) providers [34], yet no clear policy action has been taken to address absenteeism. Previous research has found that over half of physicians in Peru report dual practice as of the early 2000s, with more recent research among surgeons underscoring the financial incentive to maintain a parallel private practice despite the corrosive effects on health care quality and equity [35, 36]. In our study, 1 in 10 providers could not be found for a scheduled shift within a week of verifying the shift schedule (unplanned absenteeism), and nearly the same number had switched shifts after the schedule was finalized. Provider absence undermines the delivery of care and can erode trust in health services [37]; unplanned absences and frequent schedule switches may complicate efficient organization and delivery of services. Policies that have shown some effect in reducing absenteeism such as regulation of private sector dual practice and organizational improvements like clear communication, counseling of health care providers, and incentives for improved attendance should be considered [37].

The combination of inactive time – including early departures – and personal time during shifts added up to the equivalent of 6 h out of the 36-h work week. This reflects a substantial inefficiency in the design or delivery of health services in Lima. It is difficult to compare directly to other settings given differences in study design and health system context, but studies in Kenya and the United States have documented similar levels, with between 10 and 25% of primary care nurse and doctor time spent on personal activities [28, 38]. Additional data on productivity that were not available for this study – including patients seen and value of care provided based on clinical and patient-reported outcomes – are necessary to fully assess comparative efficiency of service delivery. Assessment of productivity could also address whether some of the personal time observed might have indirect positive effects by improving team functioning or job satisfaction. Previous research has linked longer work hours to lower satisfaction with work-life balance and found that intention to emigrate from Peru was higher among providers showing signs of burn-out syndrome [39, 40]. Policies intending to enhance health system productivity in Lima should take account of the current productive time, considering the non-negligible incidence of unscheduled absenteeism and the frequency of early departures from shifts, and the uneven use of scheduled shifts for personal time.

Providers spent just under 1 h of each shift on direct patient care on average and 10–15% of their time on indirect care. Blocks of time with patients were generally short, approximately 3–5 min on average, though it is possible individual patients spanned multiple blocks. At least as much time was spent on documentation as direct and indirect care, even apart from administrative tasks, which consumed almost 10% of doctors’ time in particular. While the large proportion of time on patient records is comparable to other settings [28], in this study much of the documentation and administrative tasks were paper-based rather than electronic. Computer use was most common among midwives, with two-thirds of midwives using a computer compared to half of nurses and one third of doctors, likely reflecting the initiation of EMR within maternity care services in Peru [21]. Twenty percent of the time observed on computers was in transcription to or from paper records. We found few notable differences in time allocation in providers who used computers compared to those who did not, suggesting that implementation of the system to date does not appear to be detracting from patient care, though neither is there evidence of decreased paperwork or increased productive time. As a whole these findings underscore the incomplete integration of electronic health systems into primary care services in Lima and the continued potential for efficiency gains with appropriate interventions, such as elimination of the need for transcription.

### Study strengths and limitations

We drew a representative sample of urban primary care providers in a complex care environment, obtained a high response rate from providers, and collected a wealth of detail on individual use of time to assess time on productive work and the use of health information systems in practice. The study is limited by differential non-response by older, permanently tenured health care workers, and by small samples within shift types. Provider behavior may have changed due to the presence of the observer (Hawthorne effect) [41]; the results are likely to represent an upper bound for active time by providers to the extent this bias was present. Previous assessments of direct observation of clinicians have not identified substantial differences in providers’ time use due to observation: providers reported that observers were quickly forgotten, and comparison of direct observation to alternative methods of recording time use have not found significant difference in categories such as personal time [27, 30, 42–44]. In studies of direct observation of provider clinical actions where a Hawthorne effect has been shown, the effect attenuated over a short time [41]. While we are not able to quantify the potential Hawthorne effect in the current data, given the length of the observations in this study and the focus on time use rather than clinical actions, we have no reason to believe provider behavior would be meaningfully altered over the full assessment. Development of the WOMBAT method in Australia included comparison across multiple studies for validation [30]; as the first application of this method in Peru, this study does not provide external validation of this method – and the categories used – for application in Lima. Future efforts to confirm validity are warranted. The study and others like it in the future would be strengthened by capturing at a minimum the number of patients seen and potentially the content of care provided to enable the assessment of efficiency within the time observed.

## Conclusions

If primary care is to be redefined, it is important to understand how providers use their time, their productivity, and the value of care to patients. We found that primary level care in Lima is weakened by absenteeism and non-work activities during scheduled shifts; substantial time is dedicated to paper-based record keeping and, for doctors, administrative responsibilities. The time motion study is a feasible method to address time use in settings such as Lima and can serve as key complements to population-based surveys on patient experience [23]. Future iterations that include patient data and the patient perspective would provide greater insight into efficiency and optimizing the value of primary care for patients [45, 46].

Interventions to improve efficiency should consider provider factors, such as dual practice in the private sector, and health system traits, such as the need for transit within facilities, the functionality of the health information system, and administrative responsibilities placed on clinicians. Particularly given the recent strain on the health system in Peru from the coronavirus pandemic, exacerbated by the aging public health care workforce, interventions must be adopted to support and revitalize the health workforce. This study provides a baseline assessment of provider time in primary care facilities in Lima and identifies substantial opportunities for improved health service delivery with the existing health workforce.

## Supplementary Information

**Additional file 1:** Supplemental material. **Table S1.** Tiers of health facilities within primary care in Lima. **Figure S1.** Dimensions, categories and subcategories for the observation protocol, doctors. **Figure S2.** Dimensions, categories and subcategories for the observation protocol, nurses and midwives. **Table S2.** Population of health care providers in Lima compared to all sampled providers. **Figure S3.** Time motion observations. **Table S3.** Duration of consecutive blocks of time with patients, by provider type and shift type. **Table S4.** Specific activities observed across providers and facility types (mean), among providers on outpatient or urgent care shifts (*N* = 161). **Figure S4.** Individual working time and personal time. **Table S5.** Proportion of time spent on direct patient care and on documentation by computer users and non-users.

## Data Availability

The protocol for the study is provided in the supplementary file. The datasets used and analysed during the current study are available from the corresponding author on reasonable request.

## Abbreviations

EMR: Electronic Medical Record
FUA: Formato Unico de Atención (Single Care Form)
MOH: Ministry of Health
SD: Standard Deviation
WOMBAT: Work Observation Method By Activity Timing
