# Supplementary material for: Health care provider time in public primary care facilities in Lima, Peru: a cross-sectional time motion study
Source: BMC Health Serv Res. 2021 Feb 6;21:123. doi: 10.1186/s12913-021-06117-9 (PMC7865111; doi:10.1186/s12913-021-06117-9)
Supplement: Supplementary file 1 — Additional file 1: Supplemental material. Table S1. Tiers of health facilities within primary care in Lima. Figure S1. Dimensions, categories and subcategories for the observation protocol, doctors. Figure S2. Dimensions, categories and subcategories for the observation protocol, nurses and midwives. Table S2. Population of health care providers in Lima compared to all sampled providers. Figure S3. Time motion observations. Table S3. Duration of consecutive blocks of time with patients, by provider type and shift type. Table S4. Specific activities observed across providers and facility types (mean), among providers on outpatient or urgent care shifts (N = 161). Figure S4. Individual working time and personal time. Table S5. Proportion of time spent on direct patient care and on documentation by computer users and non-users. [file 12913_2021_6117_MOESM1_ESM.pdf]

Supplemental material

1. Study protocol
2. STROBE Checklist
3. Table S1: Tiers of health facilities within primary care in Lima
4. Figure S1: Dimensions, categories and subcategories for the observation protocol, doctors
5. Figure S2: Dimensions, categories and subcategories for the observation protocol, nurses and midwives
6. Table S2: Population of health care providers in Lima compared to all sampled providers
7. Figure S3: Time motion observations
8. Table S3: Duration of consecutive blocks of time with patients, by provider type and shift type
9. Table S4: Specific activities observed across providers and facility types (mean), among providers on outpatient or urgent care shifts (N=161)
10. Figure S4: Individual working time and personal time
11. Table S5: Proportion of time spent on direct patient care and on documentation by computer users and non-users

Consultancy: "Social Pulse: skills, perceptions and time use in the Peruvian health sector"

Project # RG-T3296

Rationale and background information

The Peruvian health system is highly fragmented within the public and private sector, The Ministry of Health is the largest provider of care, providing services to around 70% of the population. Quality of care of public health services is perceived to be low both regarding human and physical capital. Human resources in the public sector are highly regulated, from working hours, compensation schemes and other contractual characteristics. In recent years, health worker unions have succeeded in having their salaries increased with strike threats claiming real wages had been decreasing in over 20 years. However, evidence of health worker performance is lacking, making it difficult to assess the regulatory efficiency concerning human resources in the public sector. Moreover, technological changes worldwide, growth and development trends and sustainability issues are rapidly changing workplaces, both in the private and public sector. There is growing evidence to suggest that there is a demand for new job skills and the need to identify these skills, especially in complex work environments. The public health sector is challenged with adapting to technological changes while being constrained by its own regulations.

Aim of the study

The study aims to assess how health workers (doctors, nurses, and midwives) use their time during a regular work day and to assess health workers' perceptions of their own skills as well as the skills people in their positions will need to fulfill their jobs in the future.

Methods

- **Study Design**

The study will be a cross-sectional observational study.

- **Population**

We will work in Lima, the capital city of Peru, which has 1/3 of the population in the country and with a total of 369 primary health establishments managed by 4 different Health Directions: DIRIS North, DIRIS Center, DIRIS East and DIRIS south. Primary health establishments are divided into four types from lowest to highest level and number of establishments:

  - Health posts type I-1 (5)
  - Health centers type I-2 (156)
  - Health centers type I-3 (176)
  - Health centers type I-4 (32)

The Ministry of Health in Lima oversees all hospitals located in the 4 areas: 18 hospitals and 7 specialized hospitals (institutes). Our population universe will be all doctors, nurses and midwives working (regardless of the type of contract) in health centers types I-2, 1-3, I-4, and general hospitals, a total of 382 facilities.

We classify doctors, nurses and midwives as defined by Law 23536 passed in 1982. This Laq regulates the classification of health worker professionals, where 11 health professions are considered; doctors, nurses and midwives among them. The definitions of the medical, nurse and midwife professions recognized by law match those considered in the WHO classification of health workers as medical practitioners, nursing professionals, and midwifery professionals respectively.
The following table summarizes the number of facilities and distribution of health workers across health centers and hospitals in all four administrative areas:

Area	Category	Doctors	Nurses	Midwives
CENTER	I-2	34	42	30
	I-3	357	217	136
	I-4	50	24	32
EAST	I-2	44	51	39
	I-3	124	112	111
	I-4	52	32	36
NORTH	I-2	44	72	52
	I-3	192	218	145
	I-4	183	195	163
SOUTH	I-2	80	119	86
	I-3	160	115	98
	I-4	279	126	163
HOSPITALS	II-1	245	211	75
	II-2	345	358	123
	II-E	108	107	26
	III-1	3389	3190	371
	III-2	1744	2266	203

For those with full-time permanent contracts, all three professional cadres in their capacity as care providers, must work six hours a day or 36 hours a week, with a maximum of 150 hours a month. They can also fulfill day or night shifts in emergency services, hospitalization and intensive care units, which must not exceed 12 hours, or 24 hours if needed. While day or night shifts are accounted within the 150-hour limit, health professionals receive bonuses for being assigned to shifts. Doctors can work part time if they split their duties with teaching activities within the health center or hospital.

These provisions apply for health workers with full-time permanent contracts (Legislative Decree 1156), whose salaries are also regulated by law. It guarantees job security until retirement among other benefits. Health centers or hospitals offer permanent positions through open public contests, and after five years health professionals have the option to apply for promotions. Not all health professionals are hired for permanent positions and more flexible contracts are offered within each health center or hospitals at the discretion of the administrative units in charge.

Phases

a. Design of the instruments:

a.1. Design of the instrument for direct observations of the providers (time and motion study)

Given the absence of a standardized time and motion protocol for assessing health worker time allocation, an important phase of the study will include an exploration of the literature, contact with experts and the design of an instrument. We will also evaluate the best alternatives for the collection of data (e.g. app-based). This phase includes a targeted review of the relevant literature on time and motion studies to identify best practices in data collection instruments and methods. The literature review will be conducted using PubMed, LILACS, and targeted searches of multilateral organizations including the World Bank, World Health Organization, and USAID. We will focus on time and motion studies (possible synonyms: workflow, clinician observation) conducted since 2005 with an explicit goal of capturing the content and timing of health care worker activities. Based on our findings, we will develop an instrument, select the most appropriate method for collecting data and perform the programming or adaptations needed for the instrument to be applied with cellphones or tablets, if needed.

a.2. Design of the skills-questionnaire to explore perceptions of actual skills and skills needed by health providers

As above we will explore the literature in order to design the instrument. The questionnaire would last no more than 20 minutes. It would largely focus on the importance of various abilities and skills. We will adapt the questionnaire to an electronic version to apply it through cellular phones or tablets, if needed.

b. Pilot of the content of the instruments

Instruments will be tested at a randomly selected hospital and health center. Pilot testing will include post-test interviews with field workers and with interviewees as necessary regarding any concerns or confusion about the study. Pilot data will be transmitted and analyzed for coherence, consistency, and completeness. Tools will be modified as needed based on the results of the pilot study.

c. Sampling

c.1 We will conduct sampling in two stages, first selecting the health facilities and then sampling 1 of each provider type within facility; this will yield three samples (doctors, midwives, and nurses) of independent and identically distributed observations. In stage 1, we will sample from the 208 primary facilities (I3 and I4) with probability proportionate to facility distribution by tier. We will confirm that the final sample includes facilities with INFORHUS and those without. Separately, we will select two general hospitals.

In the second stage, we will request from the Ministry of Health the list of all health providers working at each health facility as well as the monthly schedule of work with basic information regarding health care worker type of contract, length working at the establishment, and similar details typically found in the National Registry of Health Personnel (INFORHUS) database. INFORHUS is an online registry in which all

institutions in the health sector must record detailed information about their employees regardless of position and type of contract. Within each sampled facility, we will confirm the schedule for the coming week with the facility manager or obtain a schedule for the week for those facilities not covered by INFORHUS. We will randomly sample two shifts from this schedule, the first as the intended observation and the second as a replacement in case a provider declines to participate, and will sample one provider of each type within the selected shifts.

We calculate sample size sufficient to provide a confidence interval of width ± 3% around the primary outcome of time spent on direct patient care. Drawing from existing literature on time-motion observations,[1–4] we estimate standard deviation in this outcome up to 10%, which results in a sample of 54 providers per tier at primary care level. The table below shows expected distribution of health facility types in the final sample.

Área	Category	Total	Sample
Lima Metropolitana	I-3	176	35
	I-4	32	19
	II y II	24	2
TOTAL		**232**	**56**

Note that we will sample 5 providers of each type within the hospitals for a total sample of 56 facilities and 192 observations.

c.2 We will additionally apply the skills-questionnaire to the directors of DIRIS (4), Health establishments (45) and Hospitals (2).

d. Recruitment and training of field workers (enumerators and supervisors)
We will recruit experienced field workers, preferably with past exposure to health work, to become our enumerators and supervisors; this may include health workers with research experience. Training will be performed during a two-week period, which will include the methodology of the study, use of cell phones/tablets for the collection and transmission of the data in real-time, ethics in research, research integrity and a practicum. We will recruit 16 enumerators and 3 supervisors. As we have done before we will recruit in the training more participants than those needed, so we could choose those with better performance and just in case have people prepared if some of the field workers fail. All the trainees will participate in the pilot phase.

e. Data collection
We will coordinate closely with the Ministry of Health and authorities for the implementation of the study to ensure minimal disruption to clinical care.

e.1 A supervisor will perform an unannounced visit to the health center on random days in order to check for absences. This will allow us to obtain unbiased estimates of health worker attendance. The study team will attempt to locate a randomly selected subset of health workers expected to be in attendance during the time of the visit and will note his or her activity if located.

e.2 One the day of the unannounced visit, the study team supervisor will confirm the shift schedule for that week and draw the shift sample for the full assessment based on times patient care is being actively provided. Consent will be obtained from the facility director prior to the visit. Consent will be obtained from the individuals sampled on the day of the visit prior to the start of observation. At the end of the observation protocol or the next day, the enumerator would apply the skills-questionnaire to each provider and to the director of the health center/hospital. That questionnaire would last no more than 20 minutes.

e.3 The supervisor will monitor the activities and productivity of enumerators corresponding to a DIRIS. They will also perform the surveys to the directors of the DIRIS. All the data will be collected with cellphones/tablets in real time; devices will include built-in range and consistency checks to ensure high data quality. The data manager will perform spot checks for data collection from each field team immediately after field visits and will notify the supervisor in cases of missing or erroneous data rates of >5%.

f. Data analysis and report

f.1 Provider skill assessment

Provider self-assessment of skills and identification of necessary skills will be summarized using descriptive statistics overall, within tier of facility, and within provider cadre. Differences between groups will be tested using the Kruskal-Wallis test for equality of distributions or Chi squared test for differences in proportions based on the selected variable type. Analysis will seek to identify the most important skills providers selected, the current capacity of providers in these areas, and any differences between perspectives based on cadre, facility tier, or other salient provider characteristics. Health facility directors will be classified as a unique group to enable consideration of their perspectives. Predictors of identification of specific skills can be assessed with multivariable regression as appropriate.

f.2 Time and motion study

Time spent per task will be summarized using descriptive statistics (e.g. mean, median, interquartile range); task predominance will be ranked within sub-groups. Outlying providers and facilities will be identified using data visualizations as well as descriptive statistics. Differences between groups such as facility tier and provider cadre will be tested using the Kruskal-Wallis test for equality of distributions or t-tests as appropriate, with multivariate regression (generalized estimating equation models to account for repeated sampling within facilities) applied as needed to control for individual covariates in identifying determinants of time use.

The final report will provide visual and quantitative descriptive results from both instruments.

Ethical Aspects

The protocol and instruments will be presented for review and approval to the ethics committee of the Universidad Peruana Cayetano Heredia. The study will be discussed with the Ministry of Health authorities to get their support to avoid having to go through each health establishment ethics approval. We will ask for an exception of approval for the first observation but will require inform consent for the direct observation of the provider by the director of the center and by the provider itself. We will require a separate consent for the skills survey.

STROBE Statement—Checklist of items that should be included in reports of ***cross-sectional studies***

	Item No	Recommendation	Page No
**Title and abstract**	1	(*a*) Indicate the study's design with a commonly used term in the title or the abstract	1
		(*b*) Provide in the abstract an informative and balanced summary of what was done and what was found	1-2
**Introduction**			
Background/rationale	2	Explain the scientific background and rationale for the investigation being reported	4-5
Objectives	3	State specific objectives, including any prespecified hypotheses	6
**Methods**			
Study design	4	Present key elements of study design early in the paper	6
Setting	5	Describe the setting, locations, and relevant dates, including periods of recruitment, exposure, follow-up, and data collection	6
Participants	6	(*a*) Give the eligibility criteria, and the sources and methods of selection of participants	7-8
Variables	7	Clearly define all outcomes, exposures, predictors, potential confounders, and effect modifiers. Give diagnostic criteria, if applicable	9-11
Data sources/ measurement	8*	For each variable of interest, give sources of data and details of methods of assessment (measurement). Describe comparability of assessment methods if there is more than one group	*9-10*
Bias	9	Describe any efforts to address potential sources of bias	7-8
Study size	10	Explain how the study size was arrived at	7
Quantitative variables	11	Explain how quantitative variables were handled in the analyses. If applicable, describe which groupings were chosen and why	11
Statistical methods	12	(*a*) Describe all statistical methods, including those used to control for confounding	12
		(*b*) Describe any methods used to examine subgroups and interactions	NA
		(*c*) Explain how missing data were addressed	11-12
		(*d*) If applicable, describe analytical methods taking account of sampling strategy	7, 12
		(*e*) Describe any sensitivity analyses	NA
**Results**			
Participants	13*	(a) Report numbers of individuals at each stage of study—eg numbers potentially eligible, examined for eligibility, confirmed eligible, included in the study, completing follow-up, and analysed	13-14
		(b) Give reasons for non-participation at each stage	15
		(c) Consider use of a flow diagram	NA
Descriptive data	14*	(a) Give characteristics of study participants (eg demographic, clinical, social) and information on exposures and potential confounders	13-14
		(b) Indicate number of participants with missing data for each variable of interest	15
Outcome data	15*	Report numbers of outcome events or summary measures	15-16
Main results	16	(*a*) Give unadjusted estimates and, if applicable, confounder-adjusted estimates and their precision (eg, 95% confidence interval). Make clear which confounders were adjusted for and why they were included	20
		(*b*) Report category boundaries when continuous variables were categorized	NA

		(*c*) If relevant, consider translating estimates of relative risk into absolute risk for a meaningful time period	NA
Other analyses	17	Report other analyses done—eg analyses of subgroups and interactions, and sensitivity analyses	NA
**Discussion**			
Key results	18	Summarise key results with reference to study objectives	20-21
Limitations	19	Discuss limitations of the study, taking into account sources of potential bias or imprecision. Discuss both direction and magnitude of any potential bias	23
Interpretation	20	Give a cautious overall interpretation of results considering objectives, limitations, multiplicity of analyses, results from similar studies, and other relevant evidence	23-24
Generalisability	21	Discuss the generalisability (external validity) of the study results	23-24
**Other information**			
Funding	22	Give the source of funding and the role of the funders for the present study and, if applicable, for the original study on which the present article is based	27

*Give information separately for exposed and unexposed groups.

Note: An Explanation and Elaboration article discusses each checklist item and gives methodological background and published examples of transparent reporting. The STROBE checklist is best used in conjunction with this article (freely available on the Web sites of PLoS Medicine at http://www.plosmedicine.org/, Annals of Internal Medicine at http://www.annals.org/, and Epidemiology at http://www.epidem.com/). Information on the STROBE Initiative is available at www.strobe-statement.org.

Table S1: Tiers of health facilities within primary care in Lima

Tier	Main services provided	Minimum staffing
I-1	Preventive health services, minor illness, community visits	Nurse, nurse technician, midwife
I-2	Outpatient and preventive health services	Doctor, nurse, nurse technician, midwife
I-3	Outpatient and preventive health services, laboratory services. Can include uncomplicated deliveries.	Doctor, nurse, midwife, nurse technician, and laboratory technician
I-4	Outpatient and preventive health services including pediatric and obstetric morbidities, laboratory services, maternity ward, emergency room	Pediatrician, gynecologist, doctor, nurse, midwife, nurse technician, and laboratory technician

Figure S1: Dimensions, categories and subcategories for the observation protocol, doctors

ACTIVIDAD			
**Categoría**	**PC-Lee**	**PC-escribe**	**Personal**
Subcategorías	Registros<br>Archivos administrativos<br>Recetas<br>Historia clínica<br>Resultados laboratorio<br>Motor búsqueda<br>Correo electrónico<br>Personal<br>Otros	Historia clínica electrónica<br>Transcribir HCE<br>Transcribe otros<br>Registro directo<br>CNV<br>Otros	Marcar asistencia<br>Comer<br>Dormir<br>Tiempo ocioso<br>Higiene<br>Baño<br>Otro
**Categoría**	**Pape-lee**	**Papel-escribe**	**Desplazamiento**
Subcategorías	Historia clínica<br>Carnets<br>Exámenes auxiliares<br>Registro citas<br>Registros - otros<br>Referencias (libros u otros)<br>Otros	Historia clínica<br>Carnets<br>Citas<br>Referencia/interconsulta<br>FUA/HIS<br>Receta<br>Solicita pruebas<br>Altas<br>Registros-otros<br>Lista de chequeo<br>Otro	Traslado del paciente<br>Dentro del EESS<br>Fuera del EESS<br>Fuera del EESS - Visita domiciliaria<br>Otro
**Categoría**	**Procedimientos**	**Búsqueda**	**Otro**
Subcategorías	Examen físico/signos<br>Tamizajes<br>Entrega alim/suplem/medic<br>Administración medicamentos<br>Electrocardiograma<br>Curación heridas<br>Pruebas rápidas<br>Atención de partos<br>Estimulación<br>Rehabilitación<br>Ecografía<br>Otro	Preparación de materiales e insumos<br>Profesional de salud<br>Personal técnico<br>Personal administrativo<br>Paciente<br>Historia clínica<br>Otro	Inactividad<br>Pausa del observador<br>Observador no en el cuarto<br>No identificados<br>Otro
		**Solo comunicación**	**Móvil**
			Motor búsqueda - consulta<br>Motor búsqueda - personal<br>Aplicaciones - consultas<br>Aplicaciones - personal<br>Llamada<br>Mensajería<br>Otros

COMUNICACIÓN			
**Categoría**	**Paciente**	**Profs de salud**	**No comunicación**
Subcategorías	Historia del paciente<br>Explicación proc/diag/trat/prog<br>Con familiar<br>Consejería/talleres<br>Llama paciente<br>Otro	Sobre labores<br><br>Coloquial	 <br>**Otros**

LUGAR			
**Categoría**	**Consultorio**	**Ambiente**	**No EESS**
		Sala Espera<br>Recepción<br>Tópico<br>Farmacia<br>Oficina<br>Pasillo<br>Aula<br>Hospitalización<br>Otro	Visita Domiciliaria<br>Lugar público

Figure S2: Dimensions, categories and subcategories for the observation protocol, nurses and midwives

ACTIVIDAD			
**Categoría**	**PC-Lee**	**PC-escribe**	**Personal**
Subcategorías	Registros<br>Archivos administrativos<br>Recetas<br>Historia clínica<br>Resultados laboratorio<br>Motor búsqueda<br>Correo electrónico<br>Personal<br>Otros	Historia clínica electrónica<br>Transcribir HCE<br>Transcribe otros<br>Registro directo<br>CNV<br>Otros	Marcar asistencia<br>Comer<br>Dormir<br>Tiempo ocioso<br>Higiene<br>Baño<br>Otro
**Categoría**	**Pape-lee**	**Papel-escribe**	**Desplazamiento**
Subcategorías	Historia clínica<br>Carnets<br>Exámenes auxiliares<br>Registro citas<br>Registros - otros<br>Referencias (libros u otros)<br>Otros	Historia clínica<br>Carnets<br>Citas<br>Referencia/interconsulta<br>FUA/HIS<br>Receta<br>Solicita pruebas<br>Altas<br>Registros-otros<br>Lista de chequeo<br>Otro	Traslado del paciente<br>Dentro del EESS<br>Fuera del EESS<br>Fuera del EESS - Visita domiciliaria<br>Otro
**Categoría**	**Procedimientos**	**Búsqueda**	**Otro**
Subcategorías	Examen físico/signos<br>Tamizajes<br>Entrega alim/suplem/medic<br>Administración medicamentos<br>Electrocardiograma<br>Curación heridas<br>Pruebas rápidas<br>Vacunación<br>Atención de partos<br>PAP/IVAA<br>Rehabilitación<br>Otro	Preparación de materiales e insumos<br>Profesional de salud<br>Personal técnico<br>Personal administrativo<br>Paciente<br>Historia clínica<br>Otro	Inactividad<br>Pausa del observador<br>Observador no en el cuarto<br>No identificados<br>Otro
		**Solo comunicación**	**Móvil**
			Motor búsqueda - consulta<br>Motor búsqueda - personal<br>Aplicaciones - consultas<br>Aplicaciones - personal<br>Llamada

			Mensajería<br>Otros
**COMUNICACIÓN**			
**Categoría**	**Paciente**	**Profs de salud**	**No comunicación**
Subcategorías	Historia del paciente<br>Explicación proc/diag/trat/prog<br>Con familiar<br>Consejería/talleres<br>Llama paciente<br>Otro	Sobre labores<br>Coloquial	**Otros**
**LUGAR**			
**Categoría**	**Consultorio**	**Ambiente**	**No EESS**
		Sala Espera<br>Recepción<br>Tópico<br>Farmacia<br>Oficina<br>Pasillo<br>Aula<br>Hospitalización<br>Otro	Visita Domiciliaria<br>Lugar público

Table S2: Population of health care providers in Lima compared to all sampled providers

A: Characteristics of all health care workers in primary care facilities in Lima, by provider cadre

	Doctor (N = 1385)	Nurse (N = 1048)	Midwife (N = 888)	Total (N = 3321)
**Age (years)**				
Mean (SD)	52.7 (10.8)	44.7 (10.0)	49.1 (9.2)	49.2 (10.7)
**Type of contract**				
Fixed term	101 (7%)	207 (20%)	106 (12%)	414 (12%)
Permanent	1209 (87%)	786 (75%)	764 (86%)	2759 (83%)
Piecework	75 (5%)	55 (5%)	18 (2%)	148 (4%)
**Gender**				
Male	799 (58%)	47 (4%)	33 (4%)	879 (26%)
Female	586 (42%)	1001 (96%)	855 (96%)	2442 (74%)
**DIRIS**				
Lima Centro	404 (29%)	241 (23%)	168 (19%)	813 (24%)
Lima Este	175 (13%)	144 (14%)	147 (17%)	466 (14%)
Lima Norte	370 (27%)	420 (40%)	308 (35%)	1098 (33%)
Lima Sur	436 (31%)	243 (23%)	265 (30%)	944 (28%)
**Facility tier**				
I-3	824 (59%)	669 (64%)	494 (56%)	1987 (60%)
I-4	561 (41%)	379 (36%)	394 (44%)	1334 (40%)

B: Characteristics of sampled health care workers in primary care facilities in Lima, by provider cadre

	Doctor (N = 95)	Nurse (N = 92)	Midwife (N = 88)	Total (N = 275)
**Age (years)**				
Mean (SD)	47.2 (11.6)	44.6 (9.7)	47.8 (9.2)	46.5 (10.3)
**Type of contract**				
Term	16 (17%)	19 (21%)	8 (9%)	43 (16%)
Permanent	61 (65%)	64 (71%)	67 (78%)	192 (71%)
Piecework	17 (18%)	7 (8%)	11 (13%)	35 (13%)
**Gender**				
Male	43 (47%)	6 (7%)	0 (0%)	49 (18%)
Female	49 (53%)	84 (93%)	86 (100%)	219 (82%)
**DIRIS**				
Lima Centro	18 (19%)	13 (14%)	18 (20%)	49 (18%)
Lima Este	20 (21%)	24 (26%)	21 (24%)	65 (24%)
Lima Norte	29 (31%)	29 (32%)	28 (32%)	86 (31%)
Lima Sur	28 (29%)	26 (28%)	21 (24%)	75 (27%)
**Facility tier**				
I-3	61 (64%)	54 (59%)	60 (68%)	175 (64%)
I-4	34 (36%)	38 (41%)	28 (32%)	100 (36%)

Figure S3: Time motion observations

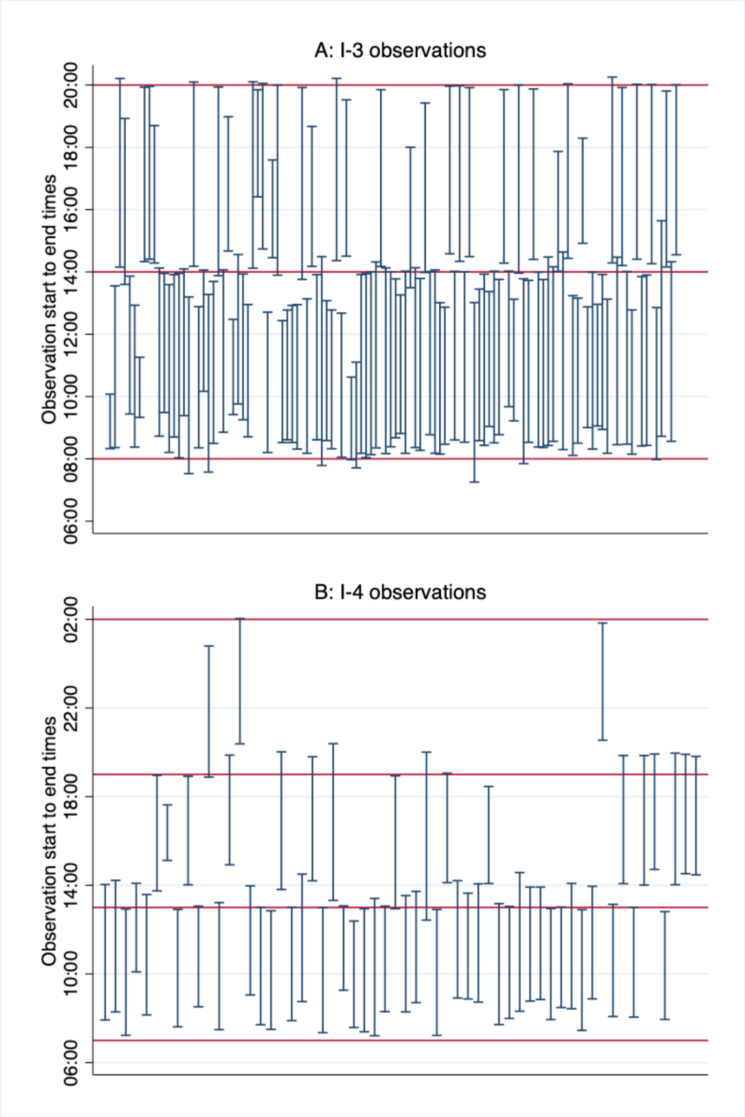

Table S3: Duration of consecutive blocks of time with patients, by provider type and shift type

	Average duration of patient-facing block (minutes)	Number of patient-facing blocks per shift
**Doctors**		
Outpatient (N=49)	3.3	34.9
Inpatient/urgent (N=7)	3.3	15.5
Other (N=1)	1.3	0.2
**Nurses**		
Outpatient (N=45)	4.3	26.3
Inpatient/urgent (N=7)	5.3	32.5
Other (N=7)	2.6	19.3
**Midwives**		
Outpatient (N=45)	4.5	30.2
Inpatient/urgent (N=8)	3.1	27.8
Other (N=3)	4.0	29.2

Table S4: Specific activities observed across providers and facility types (mean), among providers on outpatient or urgent care shifts (N=161)

Shading indicates relative percent time on activity: darker green = more time per column.

Activity type	Sub-type	Doctors Outpatient shift N = 49	Doctors Hospital shift N = 7	Nurses Outpatient shift N = 46	Nurses Hospital shift N = 5	Midwives Outpatient shift N = 46	Midwives Hospital shift N = 8
Direct care	Procedures	4.6%	2.8%	5.9%	6.8%	7.0%	8.8%
	Patient communication	10.2%	4.7%	6.2%	9.6%	7.9%	10.0%
	Other direct care	0.0%	0.0%	0.1%	12.3%	0.1%	
Indirect care	Reading files: paper	6.5%	1.5%	5.1%	2.4%	8.1%	3.7%
	Reading files: PC	0.2%	0.0%	1.2%	0.6%	0.2%	0.9%
	Preparing materials	2.6%	0.2%	6.9%	5.5%	4.1%	2.9%
	Hygiene	0.3%	0.4%	1.0%	2.1%	0.7%	1.3%
	Other indirect	3.3%	0.8%	1.9%	0.4%	4.1%	0.9%
Medication	Medication	6.4%	4.9%	2.7%	4.5%	3.5%	1.6%
Documentation	Doc: paper	11.4%	9.5%	18.5%	11.2%	18.8%	7.1%
	Doc: PC	1.6%	0.6%	3.5%	1.3%	5.7%	8.3%
Communication	Comm consults: paper	3.1%	0.4%	0.0%	0.1%	0.9%	0.3%
	Comm consults: PC	0.1%				0.1%	
	Prof communication: other	4.9%	6.9%	11.9%	8.6%	6.5%	9.6%
	Communication: other	3.1%	7.2%	1.8%	3.5%	1.4%	3.7%
Administrative	Admin: FUA	10.7%	2.4%	3.5%	0.3%	8.1%	2.0%
	Other admin	0.1%		0.0%		0.4%	1.1%
Transit	Transit	10.4%	4.6%	10.4%	10.0%	6.6%	17.9%
Education	Education	1.8%	0.0%	0.3%		0.4%	0.2%
Personal/social	Personal: eat/sleep/hygiene	2.8%	20.3%	3.3%	8.2%	3.7%	9.2%
	Personal: other	5.6%	19.7%	5.2%	10.6%	3.1%	7.0%
Inactive	Inactive	8.1%	0.8%	3.4%	0.3%	5.6%	1.7%
Unobserved	Other	2.1%	12.3%	7.3%	1.7%	3.0%	1.8%

FUA: Formato Unico de Atención (Single Care Form)

PC: Personal computer

Figure S4: Individual working time and personal time

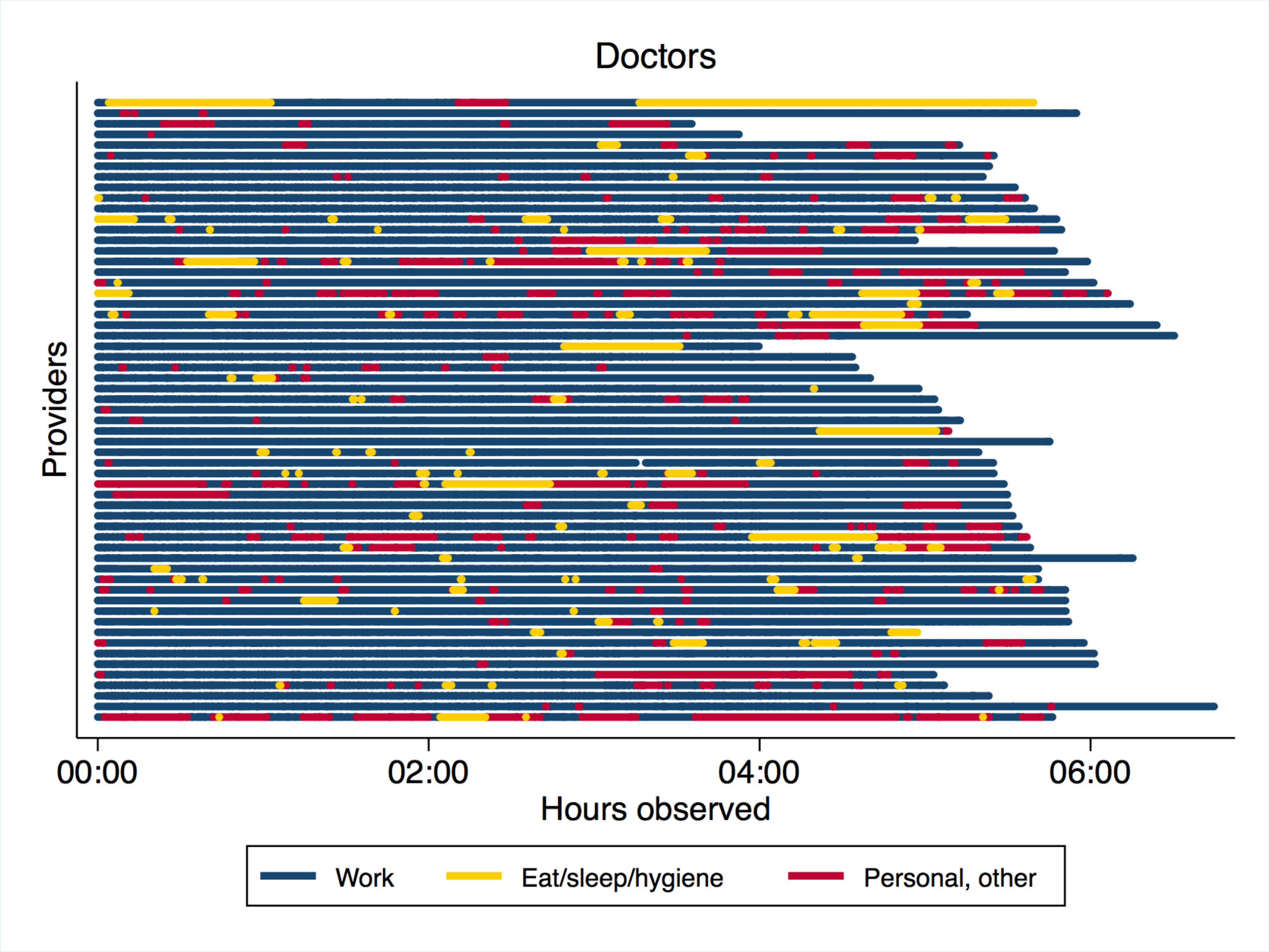

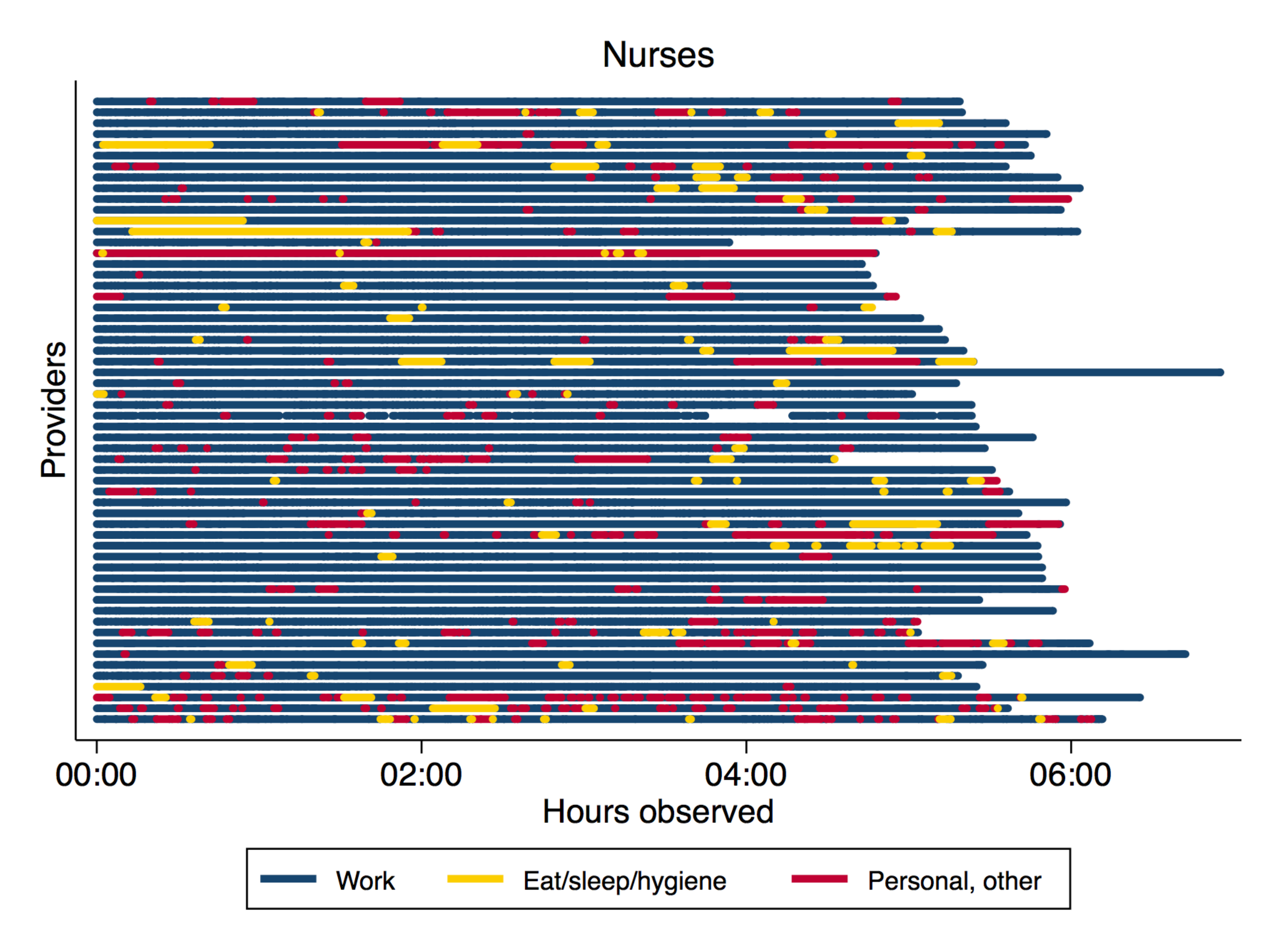

Nurses
Providers
00:00
02:00
04:00
06:00
Hours observed
Work
Eat/sleep/hygiene
Personal, other

1

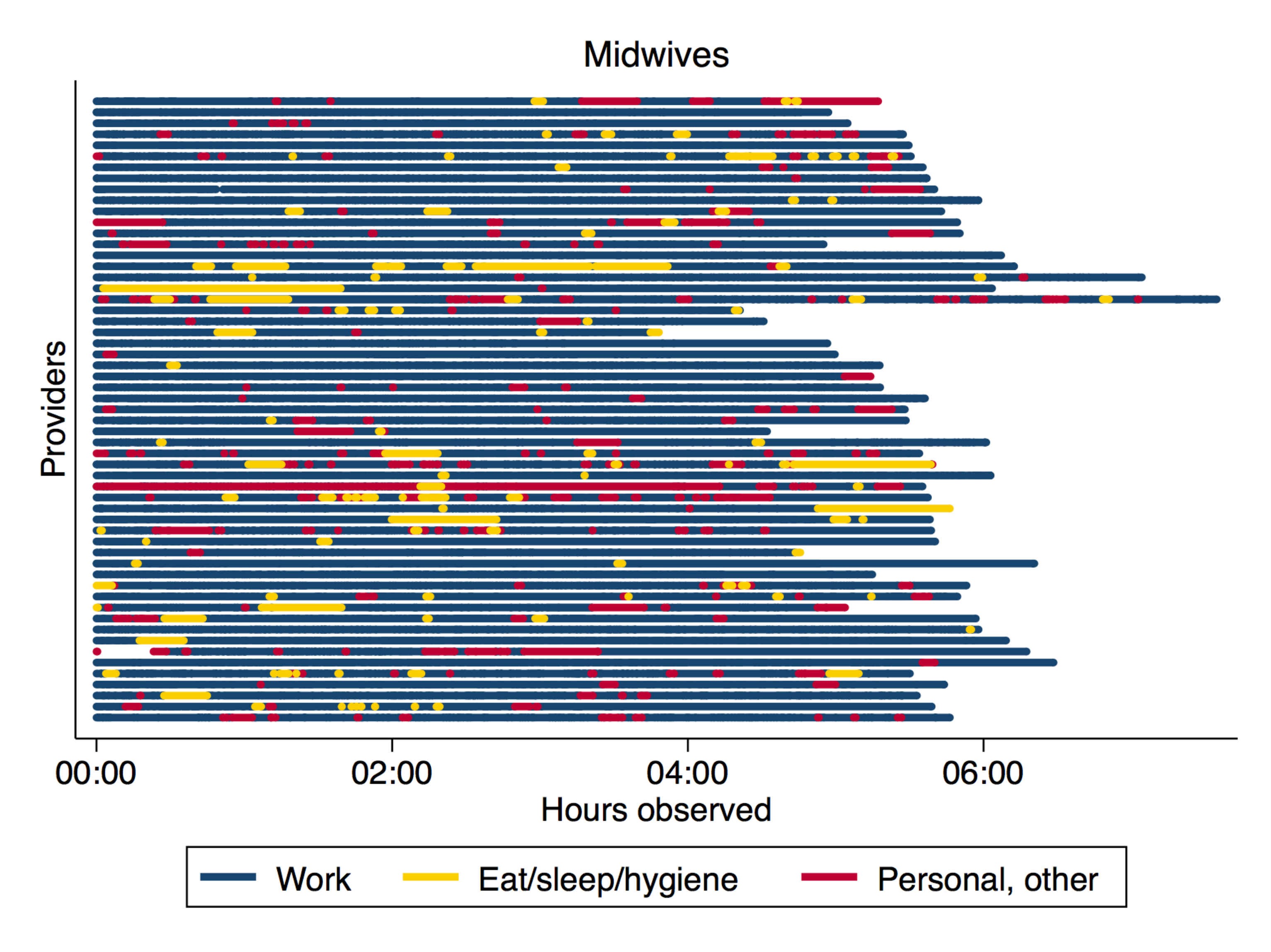

Midwives
Providers
00:00
02:00
04:00
06:00
Hours observed
Work
Eat/sleep/hygiene
Personal, other

Table S5: Proportion of time spent on direct patient care and on documentation by computer users and non-users

	Doctor No computer use (N = 35)	Doctor Computer use (N = 19)	Nurse No computer use (N = 30)	Nurse Computer use (N = 27)	Midwife No computer use (N = 14)	Midwife Computer use (N = 42)
**Direct care (proportion)**						
Mean (SD)	0.16 (0.07)	0.12 (0.07)	0.16 (0.07)	0.16 (0.15)	0.14 (0.05)	0.19 (0.11)
**Documentation (proportion)**						
Mean (SD)	0.12 (0.04)	0.15 (0.09)	0.19 (0.13)	0.20 (0.15)	0.14 (0.08)	0.26 (0.11)
